# The Tiny Companion Matters: The Important Role of Protons in Active Transports in Plants

**DOI:** 10.3390/ijms23052824

**Published:** 2022-03-04

**Authors:** Yee-Shan Ku, Sau-Shan Cheng, Ming-Sin Ng, Gyuhwa Chung, Hon-Ming Lam

**Affiliations:** 1 Centre for Soybean Research of the State Key Laboratory of Agrobiotechnology and School of Life Sciences, The Chinese University of Hong Kong, Hong Kong, China; chengsaushan@yahoo.com (S.-S.C.); sammingsin0212@gmail.com (M.-S.N.); 2 Department of Biotechnology, Chonnam National University, Yeosu 59626, Korea; chung@chonnam.ac.kr

**Keywords:** ATP-binding cassette (ABC) transporter, multidrug and toxic compound extrusion (MATE) transporter, monosaccharide transporter (MST), sucrose transporter (SUT), amino acid transporter, detoxification, nutrient transport, stress adaptation, proton gradient, cellular pH

## Abstract

In plants, the translocation of molecules, such as ions, metabolites, and hormones, between different subcellular compartments or different cells is achieved by transmembrane transporters, which play important roles in growth, development, and adaptation to the environment. To facilitate transport in a specific direction, active transporters that can translocate their substrates against the concentration gradient are needed. Examples of major active transporters in plants include ATP-binding cassette (ABC) transporters, multidrug and toxic compound extrusion (MATE) transporters, monosaccharide transporters (MSTs), sucrose transporters (SUTs), and amino acid transporters. Transport via ABC transporters is driven by ATP. The electrochemical gradient across the membrane energizes these secondary transporters. The pH in each cell and subcellular compartment is tightly regulated and yet highly dynamic, especially when under stress. Here, the effects of cellular and subcellular pH on the activities of ABC transporters, MATE transporters, MSTs, SUTs, and amino acid transporters will be discussed to enhance our understanding of their mechanics. The relation of the altered transporter activities to various biological processes of plants will also be addressed. Although most molecular transport research has focused on the substrate, the role of protons, the tiny counterparts of the substrate, should also not be ignored.

## 1. Introduction

Transporters play important roles in the transport of nutrients, hormones, and metabolites for the purposes of growth, development, and adaptation to stresses. To deliver the substrates in a specific direction, active transporters, which can transport substrates against their concentration gradients, are required. Transmembrane transporters mediate the transport of substrates across biological membranes. The polypeptides of transmembrane transporters contain transmembrane segments (TMSs), which are integrated in the membrane [1]. The segments of the integral membrane proteins embedded in the membrane are called transmembrane domains (TMDs) [2]. Such active transport is often driven by the proton gradient across the cellular/subcellular membrane. Therefore, changes in the cellular/subcellular pH could regulate the transport activities and determine the direction of transport. In each subcellular compartment, the pH is highly regulated and yet dynamic. It has been reported that stresses result in changes in cellular pH, which can form part of the stress signal [3]. For example, it was reported that flooding and drought stress induced an increase in the pH of xylem sap [3]; ionic stress induced an increase in cytosolic pH and a decrease in vacuolar pH [4]; fungal infection induced an increase in the pH of apoplastic sap; and pattern-triggered immunity (PTI) induced a decrease in cytosolic pH [5]. The changes in pH in different cellular compartments may imply the change in pH difference as well as the change in electrical potential difference across the biological membrane. Furthermore, the pH itself could also affect the activity of the transporter and the protonation state of the substrates to be transported. Altogether, these factors influence the transport efficiency and, thus, bring forth the physiological regulations.

In plants, cellular pH has been reported to be mainly regulated by proton pumps including the plasma membrane ATPase (PM-ATPase), the vacuolar-type ATPase (V-ATPase), the vacuolar pyrophosphatase (V-PPase) [6,7] and Na^+^/H^+^ antiporter (NHX) [8]. PM-ATPase hydrolyzes ATP to release H^+^, which is then exported out of the cell by PM-ATPase [9]. Such transport of H^+^ results in the proton gradient and electrical potential gradient across the membrane [9,10]. Under salt stress, it has been suggested that the PM-ATPase creates the electrochemical proton gradient to enable the extrusion of Na^+^ out of the cell or the intrusion of Na^+^ inside the vacuole by NHX [11]. V-ATPase has been known to be responsible for vacuolar acidification and create electrochemical proton gradient across the vacuolar membrane to energize substrate transport in and out of the vacuole [12,13]. In addition, V-PPase was suggested to have a higher vacuolar acidification potential compared to V-ATPase and also contribute to energize various transporters, such as Ca^2+^/H^+^, Na^+^/H^+^ and Zn^2+^/H^+^ exchangers and phosphate transporter, at the vacuolar membrane [14]. The pH of various cellular compartments is associated with the activities of proton pumps and transporters that transport substrates in exchange of H^+^.

Phytohormone transporters, alkaloid transporters, ion and ion chelator transporters, sugar transporters, and amino acid transporters are common active transporters in plants. Examples of phytohormone transporters, alkaloid transporters, and ion and ion chelator transporters include ATP-binding cassette (ABC) transporters and Multidrug Additionally, Toxic compound Extrusion (MATE) transporters, while examples of sugar transporters include MonoSaccharide Transporters (MSTs) and SUcrose Transporters (SUTs). These transporters have been reported to play important roles in various biological processes including cellular detoxification, nutrient transport, and stress adaptation. Previous reports have largely focused on the transport of substrates such as metabolites, sugars, and amino acids by these transporters. However, considering the mechanics of the transport activities, the protons required in exchange of these substrates also deserve the same attention. The awareness of the role of protons, and therefore pH, in transport activities will bring forth a more comprehensive understanding of these transporters.

## 2. Phytohormone Transporters

ABC transporters and MATE transporters are the two major types of multidrug transporters that transport various substrates including phytohormones, alkaloids, ion, and ion chelators [15].

### 2.1. Overview of ABC Transporters

The ATP-binding cassette (ABC) transporters belong to one of the largest superfamilies of transporters and are present in many organisms ranging from prokaryotes to human [16]. Driven by the free energy change associated with ATP hydrolysis, ABC transporters have been reported to drive the export or import of various substrates across biological membranes against the electrochemical gradient [17]. Based on the protein structure, ABC transporters can be classified into three structural types: the full transporter having two TMDs and two nucleotide-binding cytosolic domains (NBDs), the half transporter having one TMD and one NBD, and the third type of transporter having no TMD and two NBDs [18]. Half transporters could dimerize to form virtual full transporters [18]. The NBD of ABC transporters consists of the signature LSGGQ motif, which enables them to be distinguished from other ATPases [18]. Besides the signature LSGGQ motif, the NBD also contains other conserved motifs including Walker A, Q-loop, Walker B, D-loop, and switch H-loop [18]. The Walker A and B motifs are important for ATP binding by forming a P-loop, while the residues of Q-loop and H-loop interact with the γ-phosphate of the ATP [18].

In plants, ABC transporters were first identified as being involved in cellular detoxification by mediating the storage of xenobiotics inside the vacuole [19]. They have subsequently been reported to be involved in other biological processes such as the regulation of growth and development, tolerance to abiotic and biotic stresses, and nutrient uptake [20]. Terrestrial plants contain more ABC transporter genes in their genomes than other living organisms [21]. These transporters can facilitate the transportation of diverse substrates including hormones such as auxins and abscisic acid (ABA), and secondary metabolites such as flavonoids, reactive oxygen species (ROS), and lipid molecules [20,22,23]. Thus, ABC transporters play crucial roles in the development and survival of plants by translocating different molecules under diverse conditions. The subcellular localizations of ABC transporters in various membranes, including the membrane of peroxisome, mitochondrion, vacuole, chloroplast, and plasma membrane, have been reported [24,25,26,27,28].

#### 2.1.1. The Deprotonation of Auxin Molecules in Cytoplasm Implies the Need for Active Transporters

Auxin molecules can migrate by mass flow in the vascular system or being transported from cell to cell [29]. In the cytoplasm where the pH is near neutral, most of the indoleacetic acid (IAA) molecules exist in the anionic form, which is not favorable for diffusion across the plasma membrane [29]. Therefore, the transport of ionized auxin molecules across the membrane would need active auxin transporters [29].

It was reported that ABCB transporters are associated with the active transport of auxin driven by ATP hydrolysis [30]. *ABCB*s are mainly expressed in the leaf and root meristem and are related to the transportation of auxin in the apoplast [15]. AtABCB1 and AtABCB19 are ABC transporters mediating intracellular auxin transport and were reported to be involved in anther development [31]. The Arabidopsis double knockout mutant of *abcb1abcb19* showed reduced apical dominance, dwarfism, short hypocotyl, and poor fertility [32]. It was also reported that the direction of transport of IAA mediated by AtABCB4 could be reversed depending on the relative IAA concentration [33]. Although the substrate, IAA, was transported down the concentration gradient, such transport still had to be mediated by AtABCB4 [33]. However, the structural basis for such reverse of substate transport direction by the transport has remained unclear. In *Oryza sativa*, under salt stress, it was found that the *OsABCB* genes in leaf and root had differential expression responses towards the stress [34]. Under salt stress, a finetune of auxin levels in various plant tissues is needed to archive the balance of different biological processes such as biosynthesis and signal perception [35]. From these examples, it could be deduced that the deprotonation of auxin under stress is coupled with the regulated expression of ABC transporters to archive the finetune of auxin transports.

#### 2.1.2. The Deprotonation of ABA Molecules in the Apoplast Implies the Need for Active Transporters

ABA is involved in the signaling of both abiotic and biotic stresses [36]. One of the major roles of ABA is the regulation of stomatal opening [36]. The possible active transport of ABA between cells and between tissues has been suggested [37,38,39]. The ABA molecule is negatively charged in the cytosol where the pH is near neutral. During drought stress, the pH in the apoplast increases [40]. At a higher pH, ABA molecules undergo further proton dissociation. The negative charge of the ABA molecules implies the need for active ABA transport across membranes. AtABCG25 is an ABA exporter involved in the intercellular signaling pathway [41]. It is mainly expressed in vascular tissues and localized in the plasma membrane, suggesting the role of AtABCG25 in ABA export [41]. *atabcg25* knockout mutants exhibited enhanced ABA-sensitive phenotypes at the early growth stage compared to the wild-type [41]. On the other hand, other ABC transporters could also be ABA importers. For example, AtABCG40 is an ABA uptake transporter in the plasma membrane. Such function of AtABCG40 was demonstrated in both yeast and tobacco BY2 cells ectopically expressing *AtABCG40* [42]. Compared to the wild-type, *atabcg40* mutants had a slower uptake of ABA into the protoplast and a slower rate of stomatal closure under ABA treatment [42]. Furthermore, in wheat, Lr34, a PDR-type ABCG transporter, was found to be an ABA transporter. The ectopic expression of the *Lr34res* allele from a rice blast-resistant wheat cultivar improved the accumulation of ABA and enhanced the tolerance of rice to rice blast [43]. These examples show the important roles of ABC transporters in transporting ABA between cells, especially when ABA molecules are further deprotonated under stress.

### 2.2. Overview of MATE Transporters

Multidrug and toxic compound extrusion (MATE) transporters are antiporters that transport various molecules across a membrane in exchange for sodium ion (Na^+^) or proton (H^+^) [44]. The movement of Na^+^ or H^+^ across the membrane results in an electrochemical gradient that drives the transport of the substrates, which could be macromolecules or ions, in the opposite direction [44]. Most eukaryotic MATE transporters utilize H^+^ while prokaryotic MATE transporters can use either Na^+^ or H^+^ for this purpose [44]. In plants, MATE transporters typically consist of 12 transmembrane domains and have been reported to be located at membrane structures including the plasma membrane, vacuolar membrane, mitochondrial membrane, chloroplast envelope, and the surface of small vesicles [45].

#### The Phytohormone Transport Activities of MATE Transporters Could Be Dependent on pH or Electrochemical Proton Gradient

AtDTX50 (detoxification efflux carrier) was reported to be a plasma membrane-localized MATE-type protein for ABA efflux [46]. *AtDTX50* is mainly expressed in vascular tissues and guard cells where ABA is synthesized [46]. Under drought stress, the pH in xylem sap was found to increase from 6.1 to 6.7 [47]. Among pH 6, 7, and 8, AtDTX50 was found to be most active at pH 7 [46]. It therefore appears reasonable that, during drought, the xylem sap pH becomes closer to pH 7 to maximize the ABA-export activity of AtDTX50. However, from the mechanical perspective, it is not clear why the ABA-exporting function of AtDTX50 was highest at pH 7. Considering the near neutral cytosolic pH, when the pH of the medium was lower than 7, the proton gradient as well as the electrical potential difference across the cell membrane increased. However, such increases did not appear to enable an increased ABA export in exchange of the influx of H^+^ from the medium. It is possible that the pH itself, besides the pH gradient across the membrane, has effects on the protein function. Since MATE is not the sole transporter of ABA, it is possible that other transporters could complement the ABA transport when one transporter is not working at its optimal capacity.

Besides ABA, MATE transporter has also been reported to transport salicylic acid (SA). ENHANCED DISEASE SUSCEPTIBILITY5 (EDS5) was reported to be a chloroplast envelope-localized MATE-type protein that transports SA from chloroplast to cytoplasm [48]. The *eds5* mutant was formerly known as the *sid* (SA induction deficient) mutant [49,50]. It was found that when the proton gradient was destroyed by nigericin or m-chlorophenyl hydrazone (CCCP), the SA-exporting activity of EDS5 was disrupted [48]. Therefore, it was suggested that the SA-exporting activity of EDS5 is dependent on the electrochemical gradient generated by protons [48]. The expression of *EDS5* was induced by *Pseudomonas syringae* [51]. Upon pathogen infection, in PTI, cytosolic acidification occurs [5]. It would therefore be reasonable to speculate that, upon pathogen infection, the SA-exporting activity of EDS5 is enhanced. Altogether, the induced expression of *EDS5* and the enhanced exporting activity of the protein promote the resistance to the pathogen.

## 3. Alkaloid Transporters

### 3.1. ABC Transporter for the Storage of Alkaloids

Besides phytohormones, ABC transporter is also able to transport alkaloids. ABC transporters play an important role in vacuolar sequestration, which is an important process for minimizing the effects of toxic compounds and heavy metals [52,53]. Various secondary metabolites, including terpenoids, alkaloids and phenolics, are employed for defense purposes in plants [54]. However, the accumulation of these compounds can be toxic. Therefore, in plant cells, the secondary metabolites are either stored as the non-toxic precursors in the vacuole or exported out of the cell [55]. Berberine, a member of the alkaloid family, which is one of the largest groups of secondary metabolites in plants, was reported to accumulate in the rhizome of *Coptis japonica*, as an antimicrobial compound [56]. It was reported that CjMDR1 (*Coptis japonica* multidrug resistance 1) mediates the transport of berberine. *CjMDR1* is expressed in the xylem tissue and encodes a multidrug-resistance protein (MDR)-type ABC transporter to facilitate the transport of berberine from the root to the rhizome [56].

### 3.2. MATE Transporter for the Storage of Alkaloids

Besides exporting substrates from the cell, MATE transporters have also been shown to mediate substrate influx into organelles such as vacuoles. In *Nicotiana tabacum*, Nt-JAT1 (*Nicotiana tabacum* jasmonate-inducible alkaloid transporter 1) was reported to be a MATE-type antiporter that mediates the influx of nicotine into the vacuole in exchange of H^+^ [57]. The uptake function of Nt-JAT1 was found to be facilitated by F0F1-ATPase (F-ATPase), the ΔpH generator in the membrane, and was reduced by the dissipation of the pH gradient [57]. The lower pH inside the vacuole compared to the cytosol enabled the export of H^+^ from the vacuole. In this case, the nicotine influx into the vacuole resulted in the increase in cytosolic H^+^.

## 4. Ion and Ion Chelator Transporters

### 4.1. ABC Transporters for the Detoxification of Heavy Metal

ABC transporters have also been reported to be involved in the detoxification of various toxic heavy metals such as cadmium (Cd), mercury (Hg), and aluminum (Al). In Arabidopsis, compared to the wild-type, the double knock-out mutant *abcc1abcc2* was hypersensitive to arsenic (As) and had reduced vacuolar uptake of As(III)–PC_2_ and apoPC_2_ [26]. However, the overexpression of *AtABCC1* alone could not promote the tolerance to As [26], unless it was co-overexpressed with *AtPCS1* (*PhytoChelatin* (*PC*) *Synthase*) [26]. Using yeast as the model, it was shown that the ectopic expression of *AtABCC1* or *AtABCC2* mediated the microsomal uptake of PC_2_-As more efficiently than that of apo-PC_2_ [26]. It was therefore concluded that AtABCC1 and AtABCC2 are the transporters of PC and PC conjugates and mediate the uptake of PC-conjugated As into the vacuole for detoxification [26]. In another report, *AtABCC1* and *AtABCC2* were shown to mediate the tolerance to Cd and Hg [58]. In Arabidopsis, the double knock-out mutant *abcc1abcc2* was also hypersensitive to Cd(II) and Hg(II), and was demonstrated to be impaired in the vacuolar sequestration of Cd [58]. In addition, AtABCC1 and AtABCC2 enhanced the tolerance of PC-producing yeast to Hg(III) [58]. In rice, the ortholog of AtABCC1 and AtABCC2, OsABCC1, was also demonstrated to be a transporter that mediates the uptake of PC-As into the vacuole [59]. ABC transporters involved in mediating the tolerance to other heavy metals such as Al have also been reported and reviewed [60].

### 4.2. The Ion/Ion Chelator Transport Activities of MATE Transporters Are pH Dependent

Using *Xenopus* oocytes as the model, it was shown that a decrease in the pH of the medium favored the SbMATE (*Sorghum bicolor* MATE)-mediated efflux of citrate from the oocytes [61]. Similarly, the Arabidopsis MATE transporters, TT12 [62], FRD3 [63], and AtDTX1 [64], were also found to be substrate/H^+^ antiporters. H^+^-ATPase was suggested as the provider of H^+^ for MATE transporters in exchange for their substrates [65]. When under aluminum (Al) toxicity stress, the concerted activities of plasma membrane-bound H^+^-ATPase and a MATE transporter mediated the efflux of citrate and played a role in maintaining a stable cytosolic pH [65]. The Al-induced extrusion of citrate has been known as a strategy to enhance tolerance to Al toxicity. Under neutral pH in the cytosol, H^+^ is dissociated from citric acid from the TCA cycle [65]. The H^+^ is then transported out of the cell by H^+^-ATPase [65]. The remaining citrate is then exported from the cell by MATE, possibly with the transport of H^+^ back into the cytosol [65]. The extruded citrate molecules chelate Al cations to prevent Al cations from entering the cell [66]. This process is likely to result in the increase in cytosolic H^+^ level. It was speculated that this drop in cytosolic pH may be related to the pH regulation in the cytosol and the balancing of charges for secondary ion transports [65].

The MATE transporters DTX33 and DTX35 in Arabidopsis were reported to be tonoplast-localized MATE-type proteins that mediate Cl^−^ influx into the vacuole and regulate the vacuolar turgor [67]. The *dtx33*/*dtx35* double mutant is impaired in stomatal opening and is more tolerant to drought. Although DTX33 and DTX35 were demonstrated to function as ion channels with the measured reversal potentials in agreement with the theoretical Nernst potentials at different pH and dependent on the Cl^−^ concentration, it was also demonstrated that their activities were dependent on vacuolar pH [67]. Among vacuolar pH 5, 6, and 7, DTX33 and DTX35 had the highest activities at pH 5 [67]. However, whether the change in transport activity was due to the pH itself or the change in pH gradient, which means the change in proton gradient as well as electrical potential gradient, remains unclear.

The above examples show that the export of substrates out of the cell and the import of substrate into the vacuole by MATE transporters could mediate the upregulation of the cytosolic H^+^ concentration [57,65]. On the other hand, the differential H^+^ concentrations in different cellular compartments, which result in the pH gradient across the biological membrane where the MATE transporters are localized, regulate the activities of the transporters. Under stress, the fluctuation of cellular pH possibly plays a role to regulate the activities of MATE transporters.

## 5. Sugar Transporters

### 5.1. Classification and Structural Properties

In plants, MonoSaccharide Transporters (MSTs), SUcrose Transporters (SUTs; or SUCs, SUcrose Carriers), and Sugars Will Eventually be Exported Transporters (SWEETs) are the three major types of sugar transporters [68,69]. SUTs and MSTs belong to the major facilitator superfamily (MFS), which has the characteristic 12 transmembrane domains (TMDs), in the form of 6 N-terminal TMDs connected to 6 C-terminal TMDs via a cytosolic loop [68,70,71]. Despite having similar architectures, SUTs and MSTs could be differentiated from each other by the different domain structures [68]. On the other hand, SWEETs have seven TMDs and are characterized by an MtN3/saliva domain [68]. In terms of the transport mechanism, MSTs and SUTs are proton/sucrose symporters [72,73,74], while SWEETs are reported to be uniporters of sugars [72,73]. In the following sections, only MSTs and SUTs, whose functions are H^+^-dependent, will be discussed.

### 5.2. The SUT Family

In photosynthetic cells, sucrose is derived from the photosynthetically fixed carbon and is the major form of sugar transported via the phloem to other tissues [75,76]. SUTs are H^+^/sucrose symporters involved in loading sucrose into the phloem against the concentration gradient and are driven by the proton motive force across the plasma membrane of the sieve element–companion cell complex (SE-CCC) [77].

#### 5.2.1. The Activities of SUTs Are pH-Dependent

**Figure 1:**
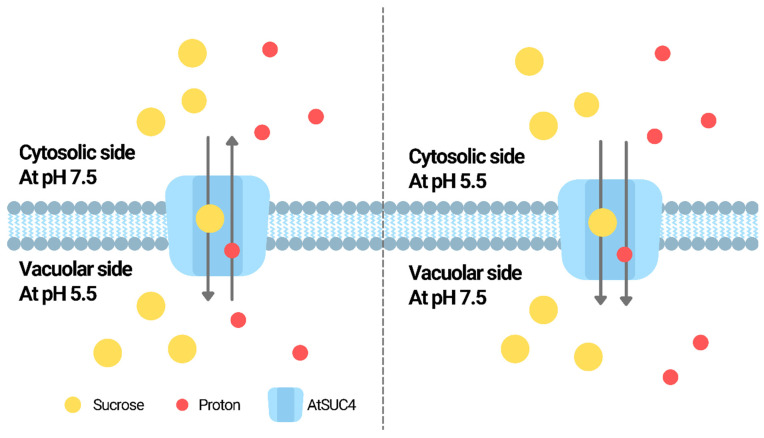
The switch of antiporter/symporter activity of AtSUC4. When the vacuole lumen was more acidic than the medium outside, AtSUC4 mediates the import of sucrose into the vacuole with the export of H^+^ from the vacuole; when the medium outside the vacuole was more acidic, AtSUC4 mediates the transport of sucrose together with proton into the vacuole [78].

The pH dependence of SUT activities has been reported in various species. For example, in Arabidopsis, AtSUC4 was reported to be a proton/sucrose symporter localized in the vacuole membrane [78]. The proton motive force was suggested to be provided by the pumping of cytosolic protons into the vacuole by V-type ATPases and V-PPases [78]. Using yeast as the model, the proton-coupled and pH-dependent uptake of sucrose mediated by AtSUT4 was demonstrated [79]. Further experiments showed that AtSUC4 could act as a H^+^/sucrose antiporter or symporter depending on the pH difference between vacuole lumen and the medium outside [78]. When the vacuole lumen was more acidic than the medium outside, sucrose was imported into the vacuole with the export of H^+^ from the vacuole; when the medium outside the vacuole was more acidic, sucrose was transported together with proton into the vacuole [78]. Such switch of antiporter/symporter activity is illustrated in Figure 1. Similar bidirectional transport of sucrose was demonstrated using the phloem-localized ZmSUT1 [80]. The sucrose/H^+^ symporter activity of ZmSUT1 was demonstrated to be pH-dependent [80]. It was suggested that both the sucrose gradient and the proton motive force determine the sucrose/proton symport direction [80].

#### 5.2.2. The Role of Sucrose Transport during Stress-Induced Cellular pH Fluctuations

*Atsuc4* mutants were found to be more sensitive to stresses including salt, osmotic, cold, and ABA treatments compared to the wild-type [81]. Probably due to the impaired sugar distribution, these mutants were found to have higher and lower sucrose, fructose and glucose in the shoots and roots, respectively, compared to the wild-type [81]. The ABA-induced expressions of stress-responsive genes, including ABA-responsive element binding factors (ABFs) and the genes upstream or downstream to ABFs, were inhibited in *Atsuc4* mutants [81]. Although the detailed mechanism remained unclear, it was suggested that AtSUC4 is involved in the crosstalk between ABA signaling and sucrose signaling [81]. The accumulation of sucrose in the root is agriculturally important for those crops that are harvested for their edible roots. In sweet potato (*Ipomoea batatas*), similar to *AtSUC4*, *IbSUT4* was found to regulate the accumulation of sucrose in the root and ABA signaling under stress [82]. The overexpression of *IbSUT4* in Arabidopsis reduced the sucrose level in the leaf but improved the sucrose level in the root under salt stress [82]. This is possibly the result of a drop in cytosolic pH induced by salt stress [83], which reduced the sucrose export from the vacuole by SUT4.

The *Phaseolus vulgaris* SUT1.1, when expressed in transgenic yeast, was found to have a higher transport activity when the pH of the medium was more acidic [84]. The tonoplast- and plasma membrane-localized PvSUT1.1 was reported to be a sucrose-proton co-transporter that is probably involved in the export of sucrose from the leaf through the phloem [84]. In the same study, the expression of *PvSUT1.1* was found to be repressed in the leaf upon heat stress [84]. Since the export of sucrose from the leaf is important for heat tolerance, the heat stress susceptibility of *P. vulgaris* was therefore hypothesized to be associated with the repression of *PvSUT1.1* under high temperature [84]. However, under high temperature in the leaf, H^+^ can leak through membranes [85]. Although not discussed in this study, such proton leaks in the cytosol may influence the transport activity of SUT in the tonoplast and plasma membrane. The net effect on the export of sucrose from the cell is unknown. To explain the SUT activity under stress, more mechanistic considerations, other than transcriptional controls, will be needed.

It has been widely accepted that SUTs are responsible for phloem loading. However, the analyses of expression data, including microarray, RNAseq, and quantitative PCR, from 167 experiments on various plant species showed that the effects on *SUT* expressions by factors such as photosynthetic rate, light level, and CO_2_ concentration are limited [86]. It was speculated that when the photosynthetic rate is increased, the existing SUT proteins may already have enough capacity to increase phloem loading without the need to increase their transcript levels [86]. Other regulatory mechanisms, such as differential intracellular localization and protein dimerization, were suggested [86]. Mechanistically, the changes in proton distribution under different environmental conditions could also potentially alter the SUT activity. Using *Amaranthus caudatus* and *Vicia faba* as the models for C_4_ and C_3_ plants, respectively, despite different kinetics and extents, the C_4_ and C_3_ plants both exhibited light-dependent cytosolic alkalization and vacuolar acidification [87]. Such alkalization and acidification peaked when the photosynthetic apparatus was maximally energized under high energy flux rates in the absence of CO_2_ [87]. Under such conditions, it is likely that the SUT activity will be affected. Therefore, the expression levels of *SUT*s under different environmental conditions could not sufficiently explain the regulation of the SUT function.

### 5.3. The MST Family

#### 5.3.1. Classification and Structural Properties

Based on the protein sequences, MSTs can be further classified into several clades, including the STP, HXT, PLT-VGT, SBG-GLT-GLUT1, ERD6-like, and TMT clades [74]. Monosaccharides including glucose, fructose, galactose, mannose, and xylose are possible substrates of MSTs [88].

Based on the crystal structure of STP10 from *A. thaliana* that has recently been resolved [89,90], it was suggested that the protein’s affinity for sugar is mainly due to the N-terminal domain and the Lid domain of the protein, while the substrate specificity is mediated by the C-terminal domain, which interacts with specific hydroxyl groups of the substrate [89]. At the apoplast, which is acidic, protonation of the Asp42 residue occurs and finetunes the protein structure to enhance the binding affinity to the substrate [89]. The subsequent unloading of glucose then enables a modification in the protein structure, which results in the release of the proton from Asp42 [90]. Since the important functional domains for the proton-coupled substrate transport are conserved among STPs, it was suggested that this transport model explains the general mechanism of action of the transporter [89,90].

#### 5.3.2. The Activities of MSTs Are pH-Dependent

In *A. thaliana*, AtPLT5 (polyol transporter 5) was reported to be a plasma membrane-localized MST-like protein that mediates the transport of sorbitol, glucose, galactose, ribose, xylose, mannitol, glycerol, and inositol [91]. Using transgenic *Xenopus* oocytes as the model and glucose and glycerol as substrate examples, the transport activity of AtPLT5 was demonstrated to be pH-dependent [91]. Maximal transport activity was demonstrated at pH 5.5 [91]. At pH 6.5, the transport activity was reduced; at pH 7.5, there was no transport activity [91]. When the extracellular pH was brought back to 5.5, the transport activity resumed [91]. AtPLT5 has a broad spectrum of substrates and is found to be expressed in various tissues [91]. Therefore, it is proposed that AtPLT5 is possibly involved in the retrieval of sugars from the apoplast [91]. The pH-dependent sugar/proton symporter activity of STPs was also reported in apple (*Malus domestica*) [92]. MdSTP13a was reported to be the transporter of both hexose and sucrose competitively to provide the sugars for pollen tube growth [92]. Using transgenic yeast as the model, optimal glucose or sucrose uptake by MdSTP13a was established at pH 6 [92]. An increase or decrease in the pH resulted in declined transport activity [92]. Different STPs have different optimal pH for their transport activities. For example, DgSTP1 from *Datisca glomerata* has the optimal pH for transport activity at pH 4.5 [93]. In plants, the transport of sugars is a major strategy to distribute or store nutrients [77]. Since different cellular compartments have different pH, understanding the pH dependence of the activities of sugar transporters is essential for the interpretation of the biological functions.

#### 5.3.3. Paralogs of MSTs Have Differential Expression Patterns to Serve Different Functions

In *A. thaliana*, *AtSTP1* was found to have a consistently high expression level in both the root and leaf among all 14 of the *STP*s identified, under normal conditions [94]. However, in the root, the expression of *AtSTP13* was highly inducible by salinity and ABA treatment [94]. Although the expression of *ATSTP1* in the root was also induced by salinity, the fold change is much less than that of *AtSTP13* [94]. Both *stp1* and *stp13* mutants had reduced abilities to uptake glucose and fructose, while *stp1* also had reduced galactose uptake [94]. After salt treatment, the leak of glucose from the *stp13* mutant was enhanced [94]. Based on the expression data and the sugar flux data, it was suggested that AtSTP13 mediates the reabsorption of monosaccharides leaked from damaged cells under salt stress while AtSTP1 is the major contributor of monosaccharide uptake under normal conditions [94]. Such differential functions of STPs in the same species are in line with another expression study on *STP*s in *O. sativa*. In this study, the expressions of *STP*s were found to be responsive to stresses including cold, high temperature, and submergence [95]. However, the patterns of expression upon the same stress were diverse among various *STP*s, which also had different expression patterns in different tissues [95].

## 6. Amino Acid Transporters

Plants can absorb inorganic and organic nitrogen from the environment via the root system [96]. In soil, inorganic nitrogen is usually found in the forms of nitrate and ammonium, while organic nitrogen usually exists in the forms of free amino acids, urea, and short peptides [97]. The uptake of nitrogen-containing molecules by plants is mediated by the respective transporters of these molecules, especially amino acid transporters, which have been known to play a major role in distributing nitrogen throughout the whole plant [98]. Amino acid accumulation and signaling have been suggested to play important roles in stress responses.

### 6.1. Classification and Structural Properties

Amino acid transporters are found in diverse plant species. They are categorized into three major families: the Amino acid Transporter Family (ATF) (which is also known as the Amino Acid/Auxin Permease (AAAP) family), the Amino acid-Polyamine-organoCation (APC) family and the Usually Multiple Acids Move In and out Transporter family (UMAMIT) [99,100,101,102]. The ATF family consists of eight subfamilies: general Amino Acid Permeases (AAPs), Lysine and Histidine Transporters (LHTs), Proline Transporters (ProTs), γ-Aminobutyric acid Transporters (GATs), aromatic and neutral amino acid transporters, indole-3-acetic acid transporters (AUXs), amino acid transporter-like proteins and Vesicular Aminergic-Associated Transporters (VAATs). The APC family consists of three subfamilies: Cationic Amino acid Transporters (CATs), Amino acid/Choline Transporters (ACTs), and Polyamine H^+^-Symporters (PHSs). Members of the ATF family and the APC family usually share similar transport activities and protein structures. These transporters usually function by one of these mechanisms: solute-cation symport, solute-solute antiport, or facilitated diffusion at the plasma membrane [103], and they have a common protein structure with 10–14 TMDs [103,104]. UMAMITs were more recently identified compared to the ATF and APC families. These transporters belong to the nodulin-like gene family, functioning as the bidirectional facilitator for amino acid transport [102,105].

### 6.2. Amino Acid Transporters Are Driven by Proton Motive Force

Amino acid transporters act as secondary active transporters, with the specific amino acid being transported coupled to the proton motive force generated by the primary active H^+^-pumping complex featuring a proton-pumping ATPase at the membrane [9,100,106,107,108]. The majority of amino acid transporters characterized in plants are proton-amino acid symporters [9,100,106,107,108]. During amino acid import, a transient alkalization of the extracellular medium was observed [106]. In transgenic yeasts expressing Arabidopsis amino acid transporter genes such as *AAP3*, *AAP4*, or *AAP5*, the proline uptake rate was increased when the external pH was made to decrease. In other words, the higher external H^+^ concentration leads to a stronger transportation driving force for the uptake of the amino acid [108]. The requirement for the driving force provided by the proton gradient was further evidenced by the amino acid transport being abolished upon the addition of the protonophore, CCCP, a compound used for disrupting the proton gradient across the mitochondrial membrane by increasing its permeability to protons [106,108,109,110,111].

### 6.3. The Expressions of Amino Acid Transporters Are Stress-Responsive

Changes in the expressions of amino acid transporters were reported under abiotic stresses such as salt and water stress in Arabidopsis, rice, wheat, and barley [112,113,114,115,116]. Spatiotemporal differences in the induction patterns of different amino acid transporters upon stress were often observed within the same plant species or among their functional homologs. The *Hordeum vulgare* (barley) proline transporter, HvProT, was suggested to be crucial in transporting proline to the root tip region upon salt stress [116]. The *Triticum aestivum* (wheat) proline transporter 3, TaProT3, was upregulated in the root under salt stress whereas TaProT1 and TaProT2 were downregulated [115]. Through genome-wide identification and evolutionary expression analyses on wheat amino acid transporters, numerous stress- and hormone-responsive *cis*-regulatory elements were found within the promoter regions of the amino acid transporter genes [117]. Similar *cis*-regulatory element analyses were conducted between *Brassica napus* (rapeseed) and Arabidopsis, and the results were consistent with previous research [117], where multiple possible transcription factor recognition sites were discovered in the promoter regions of these transporter genes [118]. Based on the gene expression data, both TaAATs and BnAAPs responded differently to different abiotic stresses, suggesting the involvement of different interconnected regulatory networks of transporters in response to specific stresses [117,118].

### 6.4. Amino Acids as Osmolytes and Their Involvement in Ion Transport Mechanisms during Stress Responses

The accumulation of proline is widely known to protect plants from water-related stresses such as salinity, drought, and freezing [119]. The high solubility of proline and low inhibitory effect on seed germination make it a good candidate as a non-toxic osmolyte. One of the toxic effects of salt stress is the mineral nutrient imbalance brought forth by the over-accumulation of Na^+^ and Cl^−^, and the reduced levels of other essential ions such as K^+^ and Ca^2+^ [120]. In different plant species, including *Olea europaea* [121] and *Cucumis sativus* [122], it was reported that the application of proline promoted salt tolerance. In *O. europaea*, it was shown that proline treatment reduced the level of Na^+^ but enhanced the level of Ca^2+^ in the leaf under salt treatment [121]. In *C. sativus*, it was shown that proline treatment reduced the level of Cl^−^ in the leaf under salt treatment [122]. Exogenous proline and glycine betaine application were also reported to relieve the inhibition on both root and shoot growth of barley seedlings due to NaCl [123]. These observations suggest a possible involvement of amino acids, such as proline and glycine betaine, in the ion transportation mechanism.

### 6.5. Amino Acid Accumulation and Salicylic Acid (SA) Signaling

Plants carry a readily utilizable form of nitrogen. It is conceivable that the amino acid composition in plants was one of the determinants of plant–pathogen interactions. The importance of amino acid transporters in sustaining pathogen growth is not surprising. For example, there are a large number of transporter protein-encoding genes in the *Psuedomonas syringae* genome [124]. The cellular concentrations of amino acids, the uptake of inorganic nitrogen, and the relocation of amino acids might contribute to the plant’s susceptibility towards the pathogen [125,126]. It has been suggested that amino acid-derived signaling can regulate SA accumulation and signaling [127,128]. A broad-specificity, high-affinity AAP homolog, *Lysine Histidine Transporter 1* (*LHT1*), expressed in the rhizodermis and mesophyll in Arabidopsis [111] was related to the pathogen susceptibility exhibited by the plant. The knockout Arabidopsis mutant *lht1* had reduced susceptibility to the hemibiotrophic pathogen, *P. syringae* [125]. It was hypothesized that the cellular redox status is modulated by nitrogen metabolism, where glutamine deficiency plays an important role in enhancing the defense responses of *lht1* plants. By studying the accumulation patterns of H_2_O_2_ and NO in the mesophyll and the spatial expression patterns of the pathogen-induced *LHT1*, it was suggested that LHT1 helped to maintain a low reactive oxygen species (ROS) level by keeping the glutamine level high. As a result, the low ROS level hindered the activation of SA-mediated defense responses [125].

In another study, Arabidopsis overexpressing *AtCAT1* (*Cation Amino acid Transporter 1*) incorporated lysine at a higher rate and was more resistant to *P. syringae* compared to the wild-type and the *cat1* mutant [126]. In the *AtCAT1* overexpressor, the resistance to the bacterial pathogen *P. syringae* was enhanced with an increased SA level in the leaves [126]. Therefore, the expression levels of amino acid transporters might affect biotic stress responses by manipulating the cellular amino acid concentrations [125,126].

### 6.6. Amino Acid Transporters Are Involved in the Regulation of Cellular pH and Rhizospheric pH

Although amino acids usually carry charges and their transportation across membranes is often coupled with protons, there was no significant direct correlation among amino acid concentrations, transport, and cellular pH [97,129]. Nevertheless, there have been reports of amino acid assimilation and transportation altering pH homeostasis in plants. Ammonium, nitrogen, and nitrate assimilation produce protons that affect the cellular pH [130]. Aspartic acid and glutamic acid are suggested to be involved in balancing the excessive H^+^ production during nitrogen assimilation [131]. Moreover, it was reported that the aluminum-activated malate transporter in wheat, TaALMT1, promoted the acidification of an alkaline rhizosphere by facilitating the exudation of both malate and the zwitterionic buffer, gamma-aminobutyric acid (GABA) from the plant root [132]. The expression level of *TaALMT1* was positively correlated with the growth performance of the wheat plant in the alkaline environment. The expression pattern of the amino acid transporter *SlCAT9* in *Solanum lycopersicum* was linked to the exchange of GABA for glutamate and aspartate during fruit ripening, and eventually led to changes in the amino acid composition of the developing fruit [131]. The result is a change in the flavor and the nutritional value of the fruit [131]. The correlation between GABA concentration and low cytosolic pH during the early stages of fruit development was demonstrated using nuclear magnetic resonance (NMR) [133]. It was suggested that the export of GABA out of the vacuole in exchange for the import of glutamate or aspartate could serve to counterbalance the proton charges through the ‘reverse’ GABA shunt pathway [131].

### 6.7. Protons Are the Unneglectable Regulators of Active Transporters under Stresses

The activities of active transporters have been reported to be pH-dependent. Such property implies the regulation of the transporter activities at biological membranes between various cellular compartments with different pH. An obvious example is the increase in proline uptake by amino acid transporters when the extracellular pH was made to decrease [108]. In addition, the different pH of the vacuole, cytosol, and apoplast further implies the finetune of the transport direction to achieve different purposes. For example, the more acidic vacuole compared to the cytosol favors the storage of toxic nicotine in the vacuole [57] and the influx of Cl^−^ into the vacuole for turgor regulation [67], while the acidification of the rhizosphere favors the exudation of malate and GABA from plant cells to the rhizosphere by TaALMT1 [132]. The pH dependence properties of active transporters even allow bidirectional transport H^+^. For example, AtSUC4 was demonstrated to have reversible direction of H^+^ transport when the H^+^ gradient between the membrane was made to reverse, although the structural basis of such reverse transport direction by the transporter has remained unclear [78]. Reverse transport direction has been observed in other transporters. The possible mechanisms underlying the reverse transport direction include the existence of two discrete conformation states of the transporter protein, which allows the binding of the substrate to either side of the transporter, and the existence of the substrate binding site, which is close to both sides of the transporter protein [134]. For example, based on the protein crystal structure, different conformations of BetP, a betaine transporter, were observed [135]. The existence of both the outward-facing conformation and the inward-facing conformation underlies the possibility of alternating access of the substrate [135]. In another study, it was reported that Glt_ph_H7 has the substrate binding site close to both sides of the transporter to allow alternating access of glutamate from either side of the transporter [136]. However, the structural basis of the revere transport direction by the transporters discussed in this review has remained unknown. More mechanical and structural studies on the transporters will be needed to address this phenomenon.

The pH of different cellular compartments is highly regulated and yet highly dynamic, especially when under stress. The alteration in pH of different cellular compartments under stress implies the regulation of transporter activities. For example, under drought stress, the pH in xylem sap was found to be increased from 6.1 to 6.7 [47], which is closer to pH 7, the optimal pH for the transport activity of AtDTX50 [46]. The significance of such pH-dependent activity lies in the possibility of improving ABA transport activity under stress. In addition, under stress, the deprotonation of phytohormones such as auxin and ABA molecules highlights the necessity of active transporters when the diffusion across membranes is made more difficult. Such reliance on active transporters for phytohormone transportation allows a highly controlled transport of the phytohormones under stresses.

Considering the mechanics of the active transporters under stress, it could be deduced that the altered expression of the transporters under stresses could not fully explain the regulation on the transport capacities. In some cases, the altered transporter activity under stress could be coupled with the altered expression of the gene encoding the transporter to improve the transport efficiency. For example, the pathogen-induced expression of *EDS5* is coupled with possible pathogen-induced cytosolic acidification [5,48,51], which favors the SA-exporting activity of EDS5. However, in some cases, the expression of transporters could not sufficiently explain the altered substrate transport under stress. For example, it was found that photosynthetic rate, light level, and CO_2_ concentration had limited effects on the expressions of *SUT*s [86]. However, an improved sucrose transport upon the increased photosynthetic rate is expected. In this case, the possible alteration of the sucrose transport efficiency could be the explanation. It is therefore important to understand the mechanics of the active transporters under different situations when the cellular pH fluctuates.

The modulation of the expression of active transporters in plants has been considered a common approach to modulate the accumulation of desired metabolites [137]. Since protons play important roles in regulating active transports in plants, attempts to modulate the transporter activities could be broadened to consider the manipulation of genes that regulate the proton levels in various cellular compartments. Such manipulation allows the finetune of transporter activities under stress when cellular pH fluctuates.

The movement of H^+^ across the biological membranes results in electrochemical proton gradient, which means the proton gradient as well as the electrical potential gradient across the membrane, to energize the substrate transport. In the above discussion, several proteins, such as AtEDS5 [48,51], AtSUC4 [78,79,81], and AtLHT1 [111,125], were reported to have their activities dependent on the proton gradient. Such observations are in line with the suggestion that the active transporters are energized by the electrochemical proton gradient across the biological membranes. In several examples, such as AtDTX50 [46], AtDTX33 [67], AtDTX35 [67], PvSUT1.1 [84], AtPLT5 [91], MdSTP13a [92], and HvProT [116], the transport activities were shown to be different under different pH of the vacuole/medium. However, it is not clear whether such differences of activities are merely a result of the altered electrochemical proton gradient across the membrane or a result of the structural change of the protein under different pH. Detailed studies on the conformation of the proteins under different pH will be needed to address the question.

Examples of the activities, pH dependence, and the biological significance of ABC transporters, MATE transporters, MSTs, SUTs, and amino acid transporters are summarized in Table 1.

## 7. Conclusions

ABC transporters, MATE transporters, MSTs, SUTs, and amino acid transporters are involved in the transport of ions, toxic compounds, sugars, hormones, and amino acids. The transport of these molecules is important in the growth, development, and stress adaptations of plants. Since these transporters are driven by proton motive force, the activities of these transporters are highly regulated by cellular pH, which is usually influenced by both abiotic and biotic stresses. Besides directly participating in the transport activity by providing the proton motive force, pH also influences the charges of the substrates to be transported. In addition, the pH itself could possibly affect the transporter function. These factors together affect the transport efficiency. The role of the proton in molecular transports deserves more attention than it has received thus far. In-depth structural and mechanical studies will be needed to delineate the effects of pH, proton gradient, and electrical potential gradient on the transporter functions.

## Figures and Tables

**Table 1:** Examples of the activities, pH dependence, and the biological significance of ATP-binding cassette (ABC) transporters, multidrug and toxic compound extrusion (MATE) transporters, monosaccharide transporters (MSTs), sucrose transporters (SUTs), and amino acid transporters.

Transporter Type	Transporter Name	Transport Activity	pH Dependence	Biological Significance	References
MATE	Nt-JAT1	Mediates nicotine influx into the vacuole	Makes use of pH gradient across vacuolar membrane, with pH inside vacuole lower than in cytosol	Storage of toxic compound	[57]
	AtDTX50	Mediates ABA efflux under drought stress	Most active at pH 7 when compared among pH 6, 7, and 8	Promotes ABA efflux under drought stress when the pH of the xylem sap becomes closer to neutral	[46]
	AtDTX33	Mediates Cl^−^ influx into vacuoles; mutant impaired in stomatal opening	Most active at vacuolar pH 5 when compared among vacuolar pH 5, 6, and 7	Promotes Cl^−^ influx into the vacuole, which is more acid than the cytosol, for turgor regulation	[67]
	AtDTX35	Mediates Cl^−^ influx into vacuoles; mutant impaired in stomatal opening	Most active at vacuolar pH 5 when compared among vacuolar pH 5, 6, and 7	Promotes Cl^−^ influx into the vacuole, which is more acid than the cytosol, for turgor regulation	[67]
	AtEDS5	Mediates SA efflux from chloroplast to cytoplasm	The transport activity is driven by the proton gradient across the biological membrane	The efflux of SA from chloroplast to cytosol promotes stress tolerance	[48,51]
SUC	AtSUC4	Mediates vacuolar sucrose storage	Acts as a H^+^/sucrose antiporter or symporter depending on the pH difference between vacuole lumen and the medium outside	Facilitates sugar distribution under stress; compared to the wild-type, mutants have higher and lower sucrose, fructose and glucose in shoots and roots, respectively, and are more sensitive to salt, osmotic, cold and ABA treatments	[78,79,81]
	PvSUT1.1	Exports sucrose from leaf through phloem	Higher activity at lower pH in medium	Involved in sucrose translocation between different tissues of plant, the downregulated expression is possibly associated with the heat susceptibility of the plant	[84]
MST	AtPLT5	Mediates transport of a large spectrum of polyols	Maximal transport activity at pH 5.5; activity reduced at pH 6.5 and no activity at pH 7	Proposed to be involved in the retrieval of sugars from the apoplast	[91]
	MdSTP13a	Mediates transport of a hexose and sucrose for pollen tube growth	Optimal uptake at pH 6 in yeast model	Growth and development	[92]
	AtSTP1	Inducible by salinity but mainly involved in the distribution of monosaccharides under normal conditions; mutant with reduced ability to uptake glucose, fructose and galactose	unknown	Growth and development, adaptation to the environment	[94]
	AtSTP13	Inducible by salinity and ABA treatments; involved in the reabsorption of monosaccharides leaked from damaged cells; mutant with reduced ability to uptake glucose, fructose and galactose	unknown	Growth and development, adaptation to the environment	[94]
ATF	HvProT	Proline transportation during salt stress	pH-dependent; the proline uptake activity o yeast mutant complemented with HvProT was the highest at pH 4.5 among pH 4.5, 5.5, and 6.5	Adaptation to the environment	[116]
AAP	AtLHT1	Transports a broad spectrum of amino acids; knockout mutant with reduced susceptibility to *P. syringae*; suspected to be involved in SA pathway	pH gradient dependent	Resistance to biotic stress	[111,125]
APC	AtCAT1	Lysine incorporation; overexpressor more resistant to *P. syringae* with increased SA level in leaves	unknown	Resistance to biotic stress	[126]
	SlCAT9	Exchange of GABA for glutamate and aspartate during fruit ripening	The transport of GABA has been suggested to play a role in regulating cytosolic pH	Growth and development	[131]
ABC	AtABCB1	Transports auxin; double mutant with *Atabcb19* resulted in developmental problem with poor fertility	unknown	Growth and development	[31]
	AtABCB19	Transports auxin; double mutant with *Atabcb1* resulted in developmental problem with poor fertility	unknown	Growth and development	[31]
	AtABCG25	Exports ABA; mutant with ABA-sensitive phenotype at early growth stage	unknown	Adaptation to the environment	[41]
	AtABCG40	Uptakes ABA; mutant with slow uptake of ABA and insensitivity towards ABA	unknown	Adaptation to the environment	[42]
	CjMDR1	Transports berberine from root to rhizome	unknown	Adaptation to the environment	[56]
	Lr34	Transports ABA; ectopic expression in wheat enhanced the tolerance to rice blast	unknown	Adaptation to the environment	[43]
	AtABCC1	Mediates microsomal uptake of PC and PC conjugates for heavy metal detoxification in vacuole	unknown	Adaptation to the environment	[26]
	AtABCC2	Mediates microsomal uptake of PC and PC conjugates for heavy metal detoxification in vacuole	unknown	Adaptation to the environment	[26]

## Data Availability

Not applicable.
